# The Electrocortical Signature of Successful and Unsuccessful Deception in a Face-to-Face Social Interaction

**DOI:** 10.3389/fnhum.2020.00277

**Published:** 2020-07-17

**Authors:** Tobias A. Wagner-Altendorf, Arie H. van der Lugt, Jane F. Banfield, Carsten Meyer, Caterina Rohrbach, Marcus Heldmann, Thomas F. Münte

**Affiliations:** ^1^Department of Neurology, University of Lübeck, Lübeck, Germany; ^2^Department of Cognitive Neuroscience, Faculty of Psychology and Neuroscience, Maastricht University, Maastricht, Netherlands; ^3^Department of Neuropsychology, Otto von Guericke University Magdeburg, Magdeburg, Germany; ^4^Institute of Psychology II, University of Lübeck, Lübeck, Germany

**Keywords:** deception, truth, lie, EEG, ERP, contingent negative variation

## Abstract

Deceptive behavior, and the evaluation of others’ behavior as truthful or deceptive, are crucial aspects of human social interaction. We report a study investigating two participants in a social interaction, performing a deception task. The first participant, the “informant,” made true or false autobiographical statements. The second participant, the “detective,” then classified these statements as truth or lie. Behavioral data showed that detectives performed slightly above chance and were better at correctly identifying true as compared with deceptive statements. This presumably reflects the “truth bias”: the finding that individuals are more likely to classify others’ statements as truthful than as deceptive – even when informed that a lie is as likely to be told as the truth. Electroencephalography (EEG) was recorded from the informant. Event-related potential (ERP) analysis revealed a smaller contingent negative variation (CNV) preceding “convincing” statements (statements classified as true by the detective) compared to “unconvincing” statements (statements classified as lie by the detective) – irrespective of whether the statements were actually truthful or deceptive. This finding suggests a distinct electrocortical signature of “successful” compared to “unsuccessful” deceptive statements. One possible explanation is that the pronounced CNV indicates the individuals’ higher “cognitive load” when processing unconvincing statements.

## Introduction

The neural and psychological processes underlying deceptive behavior, such as lying or concealing information, and the possibility of correctly detecting such deceptions with psychological tests and electrophysiological or imaging techniques have been extensively studied over the last decades (see, e.g., Meijer et al., 2016 and Suchotzki et al., 2017, for reviews).

Initially, the polygraph test – in popular media known as “lie detector” – was used. This detects physiological indicators such as heart rate, blood pressure, respiration, and skin conductivity and tries to infer from these indicators whether or not the individual is telling the truth or lying in response to a series of questions. Subsequently, techniques directly studying the brain activity during the process of deception have been established. In particular, electroencephalographic (EEG) correlates of deceptive behavior have been of interest, since the EEG provides a high temporal resolution measure of cortical function.

Event-related potentials (ERPs), which are obtained via averaging of multiple spontaneous EEG epochs, can monitor instant cognitive processing of given stimuli. Thus, the neural processes of subjects involved in an act of deception can be studied via the ERP technique. Lying or concealing information is cognitively more challenging than “simply” telling the truth and requires a suppression of the correct representation while withholding the truthful response (Langleben et al., 2002; Walczyk et al., 2013; Blandón-Gitlin et al., 2014). Thus, it has been shown that the act of deceiving someone is associated with increased ERP latencies and error rates (Allen et al., 1992; Johnson et al., 2004; Suchotzki et al., 2015). The (additional) effort involved in deceptive behavior has been termed the “cognitive load”: The cognitive load hypothesis holds that lying is cognitively more demanding than truth telling and requires an increased integration of both working memory and long-term memory retrieval (Sporer, 2016) and that deceptive behavior therefore goes along with altered behavior and/or electrophysiological parameters.

Next to the P300, an ERP component appearing as a reaction to rare and relevant (target) stimuli that has been studied in multiple deception paradigms (see, e.g., Farwell and Donchin, 1991; Verschuere et al., 2009; Suchotzki et al., 2015), in particular the contingent negative variation (CNV) component has been investigated in deception studies. The CNV classically appears as an action-preparing ERP component in paradigms, in which a warning signal precedes the actual target stimulus (Walter et al., 1964). The CNV amplitude has been found to be pronounced in deceptive behavior, indicative of increased working memory activity and “cognitive load” (Fang et al., 2003; Dong et al., 2010; Suchotzki et al., 2015). However, others have found that the CNV significantly decreased immediately before subjects had to conceal a critical item, putatively due to a moment of distraction involved in the act of deceiving (Hira and Matsuda, 1998).

Importantly, it is not only the act of (attempted) deception but also the act of its consequences in a real face-to-face social interaction – i.e., whether the deceptive (or truthful) statement is actually convincing or not – that are of interest to us here. One might hypothesize that different neural processes may underlie a lie that is totally convincing versus a lie that is immediately recognized as deception.

However, studies examining the EEG correlates of lying or concealing information with respect to the social consequences are relatively rare. Here, we report a study which investigates two participants in a social interaction: The first participant (called the “informant”) makes – in some cases deceptive – statements, and the second participant (called the “detective”) evaluates these statements as being truthful or not. EEG is recorded from the informant, i.e., the (potentially) deceiving subject.

Statements were autobiographic statements, e.g., “I have never been to Berlin” or “I love spaghetti with gorgonzola.” The statements were equally divided in terms of factual and preferential statements – since content-dependent differences in the fMRI correlates of lying have been detected in earlier research (see also Ofen et al., 2017).

Behaviorally, we expected the detectives to perform slightly better than chance in detecting the truthfulness or deceptiveness of the informants’ statements, as suggested by preceding studies (see Bond and DePaulo, 2006, for a review, where an average of 54% correct lie-truth judgments was achieved). Importantly, with regard to the distribution between classifications-as-truth and classifications-as-lie, one could expect the detectives to be more inclined to believe a statement (to classify it as truth) than to consider it a lie. This so-called truth bias has consistently been reported (see, e.g., Bond and DePaulo, 2006, for meta-analyses) and has motivated the “truth default theory,” i.e., the view that truth-telling is the default mode of human communication and that people thus tend to presume that other people communicate honestly most of the time (Levine, 2014).

Electrophysiologically, we expected to see a CNV in the informant ERP prior to his/her binary “Yes/No” statement, indicating anticipation and response preparation. We further assumed that the CNV would be more pronounced in the lie condition, as predicted by the “cognitive load” hypothesis – the hypothesis that lying comes at “cognitive cost” and that this cost is reflected in the alteration of behavioral and electrophysiological parameters – and as observed in several previous studies (Fang et al., 2003; Dong et al., 2010; Sun et al., 2011; Suchotzki et al., 2015); although the literature is somewhat divergent, and e.g. Hira and Matsuda (1998) reported a smaller CNV for lying.

However, it is up to now unclear, whether the informant ERP differs with respect to the subsequent detective classification as truth or lie, i.e., whether convincing or unconvincing statements go along with a distinct phenotype of electrocortical activity as reflected in the ERP.

## Materials and Methods

### Participants

A total of 54 healthy participants (25 female, 29 male), aged 20–30 years (mean: 23.7), took part in the study. All participants were students from different faculties of the Otto von Guericke University, Magdeburg, recruited by email postings and ads. Participants did not know each other (self-report). Before taking part in this study, participants signed an informed consent form. The study was approved by the local Ethics Committee.

### Procedure

Two individuals participated in each session. The participants sat face to face at a table, within a distance of approximately 80 cm. In short, a proposition was presented to both participants via speakers. The first participant, called the “informant,” was instructed to make a – truthful or deceptive – binary “Yes/No” statement (by spelling out “ja” or “nein,” respectively) whether the given proposition applied to him/her (e.g., Proposition: “I have been to Berlin” – Informant: “Yes/No”). The second participant, called the “detective,” had to classify the informant’s statements as true or false.

**FIGURE 1:**
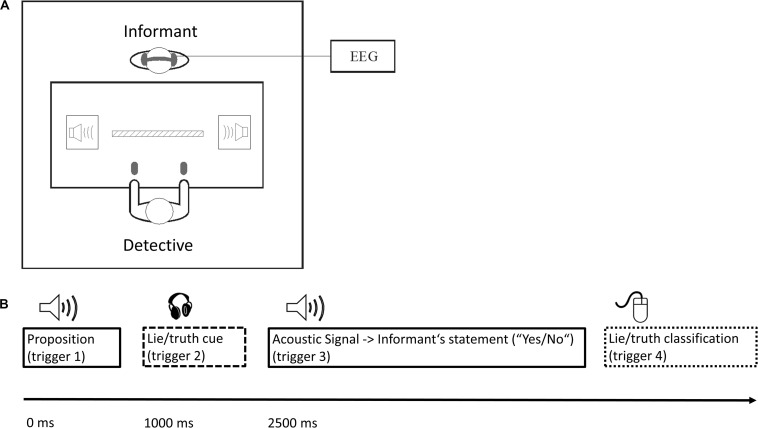
**(A)** Experimental set-up. Participants (informant and detective) sat face to face at a table, within a distance of approx. 80 cm. They could see and evaluate each other’s facial expressions, but a divider of 25 cm height in the middle of the table prevented the informant from seeing which button the detective pressed. After each block comprising 160 propositions/statements, roles were changed. The informant was continuously recorded with 32-channel EEG. **(B)** Trial procedure. The proposition was presented via speakers to both participants. After 1000 ms, only the informant received, via an in-ear headphone, the instruction to lie or to tell the truth (lie/truth cue). After 2500 ms, both participants heard the acoustic signal instructing the informant to make his or her binary (“Yes/No”) statement. Subsequently, the detective classified the statement as truth or lie by pressing a button. Dashed line: informant; dotted line: detective; solid line: both participants.

Propositions were spoken by a male German native speaker (trigger 1). After a pause of 1000 ms, the lie/truth-cue was presented to the informant via an in-ear headphone (instructing the informant to respond deceptively or truthfully, via the German words “Lüge” or “Wahrheit”; trigger 2, onset of instruction). After another pause of 1500 ms to allow the informant’s statement preparation, an acoustic signal occurred (trigger 3) and the informant made his/her binary “Yes/No” statement. Subsequently, the detective classified the statement as truth or lie by pressing one of two buttons (trigger 4). A divider of 25 cm height between the two participants prevented the informant from seeing which button the detective had pressed. Five seconds after trigger 3, the next cycle started with the presentation of the next proposition (see Figure 1 for the experimental set-up and a schematic overview of each trial).

An equal number of two types of autobiographic propositions were used: preferential statements and fact statements (e.g., “I like movies with…” vs. “I have seen the movie…”). In total, 640 propositions were presented, in four blocks. After the first block comprising 160 propositions/statements, roles were changed so that the informant became the detective and vice versa. Each block consisted of 80 different propositions that were presented twice in different randomized orders, one time combined with a “truth” cue, one time combined with a “lie” cue. One whole experimental session consisted of four blocks. Blocks 1 and 4, and blocks 2 and 3, respectively, comprised the same propositions, so that an ABBA design for the propositions and an ABAB design for the informant/detective role were implemented. Continuous 32 electrode EEG recording was carried out in the informant.

To ensure high motivation, participants were told that their reward for participating in the experiment would be between 15 and 25 €, depending on rates of “successful” deception (in the informant role) and of correctly detected statements (in the detective role). However, each participant was given 25 € reward after the experiment.

Pilot testing of the propositions was performed prior to the start of the experiment, to select propositions which were answered with “yes” in about 50% of the cases and “no” in about 50% of the cases (to prevent the detective from detecting deception based solely on predictable response probabilities); 24 subjects participated in the pilot test. They were asked to either affirm or deny each of the propositions (and in addition had the possibility to evaluate “neither yes nor know,” which lead to the exclusion of the proposition). The answer “yes” was operationalized by the number 1 and the answer “no” by the number 0. Statements that were affirmed or denied by about the same number of subjects were considered suitable for the experiment; accepted were mean values between 0.30 and 0.70.

### Data Acquisition

Informant EEG was recorded continuously from 30 unipolar tin electrodes placed according to the International 10–20 system, using an electrode cap (Electro-Cap) and a 32-channel SynAmps amplifier. Sampling rate was 250 Hz; band-pass ranged from 0.05 to 30 Hz. Electrode impedances were kept under 5 kΩ. Electrode locations were Fp1/2, Fpz, F3/4, C3/4, P3/4, O1/2, F7/8, T7/8, P7/8, Cz, Fz, Pz, FC1/2, CP1/2, PO3/4, FC5/6, and CP5/6. Reference electrodes were put on the mastoid process. Vertical electrooculogram (vEOG) and horizontal electrooculogram (hEOG) were monitored from electrodes placed below and above the eye, and at the left and right outer canthi, respectively. EEG and EOG data were recorded with Acquire^®^ software. For EEG analysis, EEGLAB (Delorme and Makeig, 2004) and ERPLAB (Lopez-Calderon and Luck, 2014) were used. EEG was segmented into 2560 ms intervals (100 ms before, 2460 ms after reference point). To remove ocular artifacts, ICA (independent component analysis) was used. To account for non-ocular artifacts such as amplifier blocking or sudden jumps in amplitude the “moving window peak-to-peak threshold” function was used, with a threshold potential individually adjusted for each participant after visual inspection of long stretches of EEG. Epochs containing these artifacts were excluded from the analysis. Three participants were excluded from ERP analysis due to a high artifact rate. Stimulus-locked informant ERPs were filtered with a 20-Hz low-pass filter. For baseline correction, baseline was defined as the interval from -100 to 0 ms. From the resulting data, averages were formed over trials for each segment and participant, and subsequently, grand averages were calculated across participants. Behavioral data of the detective (classification as truth/as lie and reaction times) was recorded with Presentation^®^ software.

### Statistical Analysis of ERP Data

Visual inspection of the informant grand average ERP waveforms revealed a broad late negative component after trigger 2 (the lie/truth cue; stimulus-locked data). This was quantified by a mean amplitude measure between 950 and 1550 ms, where visual inspection revealed a difference between the classified truth and the classified lie condition. We analyzed the electrodes Fp1/Fp2/Fpz/F3/F4/Fpz/F7/F8.

Statistical analysis was done with open source tool jamovi^®^ (Version 0.9, retrieved from https://www.jamovi.org). For ERP data analysis, repeated measures ANOVA with Huynh-Feldt correction was performed. Uncorrected *F*, but corrected *p*-values are reported. Early ERP components (N200 and P300) were not analyzed, since we cannot rule out the possibility that any differences found between conditions may be driven by the different physical properties of the acoustic stimuli used as truth (sound “Wahrheit”) or lie (sound “Lüge”) cues. Bar plots were created with GraphPad Prism (version 6, Graphpad software Inc., La Jolla, United States).

## Results

### Detective Behavioral Data

Behavioral analysis of the detective detection data showed a correct classification rate of 56.1% (SD = 4.9%), which is significantly higher than chance (50%) (*t* = 8.140, *df* = 41, *p* < 0.001, two-tailed). For the lie condition, 53.4% (SD = 5.8%) of the classifications were correct (*t* = 3.764, *df* = 41, *p* = 0.001, two-tailed); for the truth condition, 58.7% (SD = 6.4%) of the classifications were correct (*t* = 8.879, *df* = 41, *p* < 0.001, two-tailed). Detectives were significantly better at discovering the truth than at discovering a lie (*t* = 4.817, *df* = 41, *p* < 0.001, two-tailed).

Mean detective reaction times (relative to trigger 3) were significantly shorter for correct classifications compared to incorrect classifications: 2085 ms (SD = 312 ms) versus 2129 ms (SD = 294 ms) (*t* = -3.793, *df* = 41, *p* < 0.001, two-tailed).

### Informant ERP Data

**FIGURE 2:**
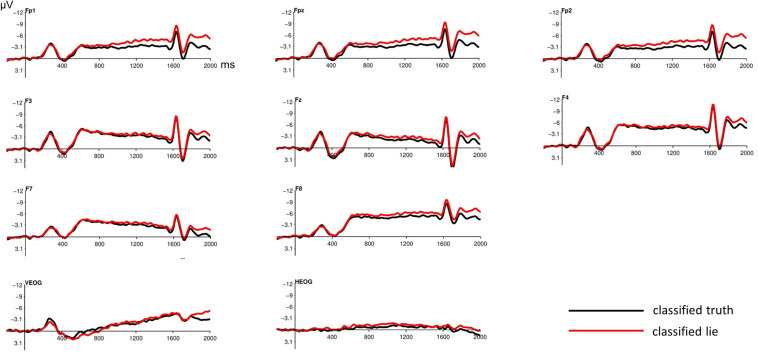
Informant stimulus-locked grand average ERP waveforms after the lie/truth cue (trigger 2) at frontopolar/frontal electrodes. The late negativity component differs between the “convincing/unconvincing” conditions: Convincing statements (classified, whether truthful or deceptive, as truth by the detective) are associated with a less pronounced negativity between 950 and 1550 ms, especially at frontopolar electrode sites. vEOG and hEOG show no contribution to effect. The potential at 1600 ms represents processing of trigger 3 (acoustic signal instructing the informant to make his/her statement). Baseline used is -100 to 0 ms. The displayed waveforms were filtered with a 20-Hz low-pass filter.

**FIGURE 3:**
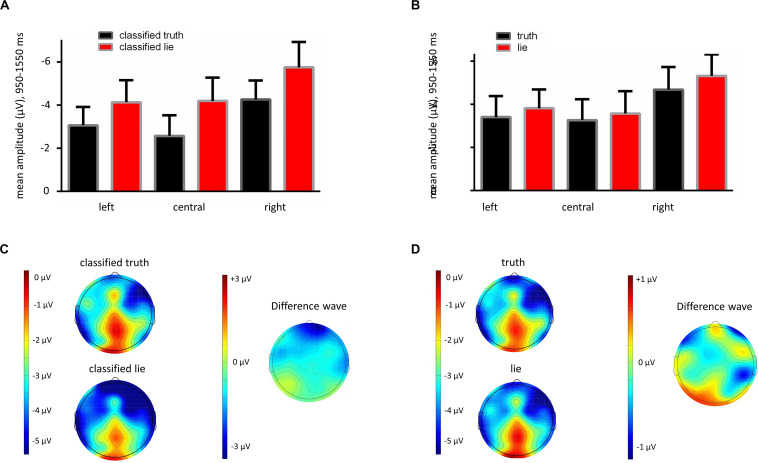
**(A)** Bar plots showing mean amplitudes at 950–1550 ms after trigger 2 (lie/truth cue) for informant frontopolar and frontal electrodes. Classified truth (“convincing”) condition depicted in black, classified lie (“unconvincing”) condition depicted in red. Note that unconvincing statements go along with a pronounced negativity component as compared to convincing statements. **(B)** Bar plots showing mean amplitudes at 950–1550 ms after trigger 2 (lie/truth cue) for informant frontopolar and frontal electrodes. Truth condition depicted in black and lie condition depicted in red. Note that no significant difference between conditions is present. **(C)** Left: Topographies at 950–1550 ms after trigger 2 (lie/truth cue), showing a difference in frontal negativity between the classified truth and the classified lie conditions. Right: Topography for difference waveform between the classified lie and the classified truth conditions. **(D)** Left: Topographies at 950–1550 ms after trigger 2 (lie/truth cue), showing no difference in frontal negativity between the truth and the lie conditions. Right: Topography for difference waveform between the lie and the truth conditions.

Significant differences in the informant ERPs were detected in the time period after the lie/truth cue (trigger 2), instructing the participants to respond truthfully or deceptively to the given proposition. The inspection of the ERPs revealed a late negative component at frontal electrode sites. Electrode positions Fp1/Fp2/F3/F4/F7/F8/Fpz/Fz were analyzed, with a 2 (classified truth vs. classified lie) × 3 (laterality: left vs. central vs. right) repeated measures ANOVA with Huynh-Feldt correction. To test for differences between conditions, the interval between 950 and 1550 ms was used (at 1600 ms, the processing of the auditory signal instructing the informant to make his/her statement, i.e., trigger 3, started). “Convincing” statements (i.e., all statements classified as truth, whether truthful or deceptive) are associated with a less pronounced negativity between 950 and 1550 ms as compared to “unconvincing” statements (i.e., all statements classified as lie) (Figures 2, 3). Repeated measures ANOVA showed a significant main effect for the classified truth (i.e., convincing) versus classified lie (i.e., unconvincing) condition for frontopolar/frontal electrodes (Fp1, Fp2, Fpz, F3, F4, Fz, F7, F8): *F*(1,40) = 4.2, *p* = 0.047, η^2^_p_ = 0.095. Differences in the vEOG channel were not found.

For the conditions lie/truth, correct/incorrect, facts/preferences, no significant differences were found, which was analyzed with a 2 (lie vs. truth) × 2 (correct vs. incorrect) × 2 (fact vs. preference) × 3 (laterality: left vs. central vs. right) repeated measures ANOVA with Huynh-Feldt correction: lie/truth: *F*(1,40) = 0.58, *p* = 0.45; correct/incorrect: *F*(1,40) = 1.81, *p* = 0.19; fact/preference: *F*(1,40) = 0.004, *p* = 0.95. The laterality effect was significant: *F*(1,40) = 6.21, *p* = 0.003 (see Figure 3 for bar plots and topographies of the lie/truth difference, and see Supplementary Figure S1A for additional ERP visualization of the lie/truth difference).

As visual inspection suggested that the classified truth versus classified lie difference was mainly driven by preference statements as compared to fact statements (see Supplementary Figure S1B for ERP visualization of the fact/preference difference), an additional 2 (classified truth vs. classified lie) × 3 (laterality: left vs. central vs. right) × 2 (fact vs. preference) repeated measures ANOVA with Huynh-Feldt correction was performed that, however, revealed no significant classified truth/classified lie ^∗^ fact/preference interaction: *F*(1,40) = 0.29, *p* = 0.60.

## Discussion

The present study examined two participants in a social interaction, performing a deception task. In each of the four blocks, one participant (the “informant”) had the role of either telling the truth or deceiving the other person by responding “Yes” or “No” in relation to a series of factual and preferential autobiographical questions. The other participant (the “detective”) tried to ascertain whether the informant was lying or telling the truth when responding to each of these questions. Each participant played the role of detective in two blocks and informant in the other two blocks. Participants did not know each other prior to the study, so they had no way of knowing the correct answers. Detectives’ behavioral responses, classification as truth or lie, and reaction times were detected, and informants’ event-related EEG potentials were recorded with 32-channel EEG. The relevant trigger for later ERP analysis was the lie/truth cue (instructing the informant via headphones to report truthfully or not).

### Detective Behavioral Data

Detectives were able to classify true and false statement slightly better than chance, probably due to subtle signs of lying given by the informant. Non-verbal behaviors such as mimic or vocalic characteristics have been, for a long time, considered as carriers of authentic messages, eventually betraying the truth when verbal communication is deceptive (Ekman and Friesen, 1969; Burgoon et al., 2020). However, as the informant in turn, too, might watch for signs of suspicion in the detective (Buller and Burgoon, 1996), and as non-verbal behaviors might be subject themselves to deceit strategies (Burgoon et al., 2020), the face-to-face social interaction between the informant and the detective incorporates a very complex pattern of verbal and non-verbal communication. Overall, the effect of correct detective classifications is rather small (56% correct classifications, with chance being 50%), which is consistent with previous studies (see, e.g., Bond and DePaulo, 2006, 2008).

Importantly, we found the detection rates to be significantly higher for true than for deceptive statements (59% vs. 53%). This is likely to reflect the “truth bias.” Truth bias theory states that individuals are more likely to classify statements of others as truthful than as deceptive (Vrij, 2000; Bond and DePaulo, 2006; Levine, 2014; Street and Masip, 2015). When truth-telling is the “default” mode of human communication, people tend to presume that other people communicate honestly most of the time (Levine, 2014). Our study shows that the truth bias even holds if participants definitely know that their counterpart tells a (instructed) lie with a 50% probability.

Detectives were found to react faster when their classification of the informant’s statement was correct. This considerably is due to the detectives being more (subjectively) certain in the case of a correct classification and therefore responding more promptly.

### Informant ERP Data

Analysis of the informant ERPs revealed a frontal late negativity that differed between the convincing and unconvincing condition: Convincing statements (i.e., a truth correctly classified as truth and a lie incorrectly classified as truth – “classified truth,” for short) were found to be associated with an attenuated frontal negativity component as compared to unconvincing statements (i.e., a correctly detected lie, a truth incorrectly classified as a lie – “classified lie”). There was a trend that this difference was driven by preference rather than fact statements. Differences in frontal late negativity between the truth and the lie condition interestingly were not found.

This late negativity component can be identified with the CNV, which appears after a warning signal preceding the actual target stimulus, indicating the participant’s action preparation (Walter et al., 1964). The early CNV is thought to be an index of cortical arousal during orienting and attention, whereas the late CNV is considered to reflect anticipation and response preparation. The CNV has been related to neural activity in prefrontal and orbitofrontal cortices, the cingulate gyrus, the supplementary motor area, as well as thalamus and bilateral insula (Nagai et al., 2004a; Bareš et al., 2007). Lateral prefrontal lesions have been shown to result in alterations in the early as well as the late CNV component (Funderud et al., 2013).

Previous studies have found a pronounced CNV during lying, which has been attributed to increased working memory activity and higher “cognitive load” in the lie condition (Fang et al., 2003; Dong et al., 2010; Sun et al., 2011; Suchotzki et al., 2015), although others have found the CNV to decrease before subjects had to conceal a critical item (Hira and Matsuda, 1998). However, much of the previous work on the EEG correlates of deception has only focused on the difference between the lie and the truth conditions and did not investigate the effect of the truthful or deceptive act in a social interaction, i.e., whether participants were convincing (e.g., in case of a “successful” lie) or not.

In the present study, we analyzed deceptive acts and their classification in an interactive paradigm. We found no significant CNV difference in the informant between the truth and the lie conditions, but a difference with respect to the subsequent (detective) classification as truth or lie: Unconvincing statements (statements classified as lie, irrespective of being truthful or deceptive) were found, in frontal electrodes, to be associated with a more pronounced CNV amplitude as compared to convincing statements (statements classified as truth). This might be indicative of the higher working memory activity and higher “cognitive load” in the classified lie, i.e., unconvincing condition, leading to a more negative frontal CNV.

It has been argued that effective intentional preparation is reflected in an increased CNV amplitude, since it is associated with faster response times and increased behavioral performance, and the CNV thus provides an index of active intentional control (Fan et al., 2007; Poljac and Yeung, 2012; Glazer et al., 2018). The magnitude of the CNV has been shown to be inversely related to sympathetic arousal (Nagai et al., 2004b). However, our study incorporated a more complex study design; two participants in a complex social interaction task involving both deception and deception-detection were investigated. Thus, signals of the informant being concentrated, calm and well prepared might have been noticed by the detective and led to a more skeptical attitude so that he or she tended not to believe the informant’s statements, i.e., tended to classify the informant’s statements as being deceptive. Importantly, the informant CNV effect reflects differences in expectation processes, not only with respect to his or her own response but also with respect to the detective’s classification: Subtle signs shown by the detective of being convinced or not convinced might be a potential source of information for the informant. Greater CNV thus might be an index of greater tension in the informant on some trials, which in turn may be noticed by the detective, leading him or her to make a “lie” decision. However, it has to be stated that the discussion about the subtle mutual influence of the two participants is very speculative and that further studies investigating face-to-face social interactions are needed.

It should be noted that the CNV observed in the informant shows a topography somewhat untypical of CNV, which has characteristically been described to have a frontocentral (not prefrontal) maximum. The activity over frontocentral electrode sites is thought to reflect activation of motor areas, i.e., motor preparation. However, as Suchotzki et al. (2015) point out, the (late) CNV has been assumed to indicate various aspects of the cognitive processes presumably involved in (attempted) deception, such as increased working memory activity, motivational aspects, and stronger outcome monitoring (see, e.g., Honda et al., 1996; Brunia and van Boxtel, 2001; van Boxtel and Böcker, 2004). Thus, one might assume to see CNV effects during a deception task not only over motor areas but also over frontal and prefrontal areas. E.g., the study by Suchotzki et al. (2015) found the deception-induced CNV effect, too, to be restricted to Fz, which is in line with our data.

In summary, the informant frontal negativity differed during statement preparation between the “convincing” and the “unconvincing” condition, whereas a difference between the “truth” and the “lie” condition was not found. This latter finding contrasts with the reported CNV enhancement during lying attributed to the “cognitive load” observed in previous studies. However, as subtle signs of the informant’s cognitive stress or “load” may have lead the detective to classify the informant’s statement as lie, this thus may provide an explanation for the pronounced CNV accompanying informant’s statements classified as lie by the detective.

To conclude, although EEG studies investigating individuals in a social interaction are technically demanding and subject to several limitations (e.g., the body movements normally associated with social interactions must be suppressed), they provide unique evidence for the neural correlates of social behavior. Future studies should further address the electrophysiology of convincing deceptive behavior and the detection of this behavior in multiple social situations.

## Data Availability Statement

The raw data supporting the conclusions of this article will be made available by the authors, without undue reservation.

## Ethics Statement

The studies involving human participants were reviewed and approved by Ethics Committee of the Otto von Guericke University Magdeburg, Magdeburg. The patients/participants provided their written informed consent to participate in this study.

## Author Contributions

TW-A, AL, JB, CM, CR, MH, and TM designed and conducted the study and data analysis. TW-A wrote the first draft of the manuscript. AL, JB, MH, and TM critically revised the manuscript. All authors contributed to the article and approved the submitted version.

## Conflict of Interest

The authors declare that the research was conducted in the absence of any commercial or financial relationships that could be construed as a potential conflict of interest.

## Supplementary Material

FIGURE S1 **(A)** Informant stimulus-locked grand average ERP waveforms after the lie/truth cue (trigger 2) at frontopolar/frontal electrodes for the truth and the lie conditions. No significant difference in CNV is present. Differences in early ERP components (N200 and P300) were not analyzed, as we cannot rule out that any differences found between conditions may be driven by the different physical properties of the acoustic stimuli used as truth (sound “Wahrheit”) or lie (sound “Lüge”) cues. Baseline used is -100 to 0 ms. The displayed waveforms were filtered with a 20-Hz low-pass filter. **(B)** Informant stimulus-locked grand average ERP waveforms after the lie/truth cue (trigger 2) at frontopolar/frontal electrodes for the classified lie versus classified truth conditions, separated for fact and preference statements. Visual inspection suggests that the classified truth versus classified lie difference is driven by preference rather than fact statements, which, however, is not statistically significant. Baseline used is -100 to 0 ms. The displayed waveforms were filtered with a 20-Hz low-pass filter.
