# Supplementary figures and images for: The Electrocortical Signature of Successful and Unsuccessful Deception in a Face-to-Face Social Interaction
Source: Front Hum Neurosci. 2020 Jul 17;14:277. doi: 10.3389/fnhum.2020.00277 (PMC7379373; doi:10.3389/fnhum.2020.00277)

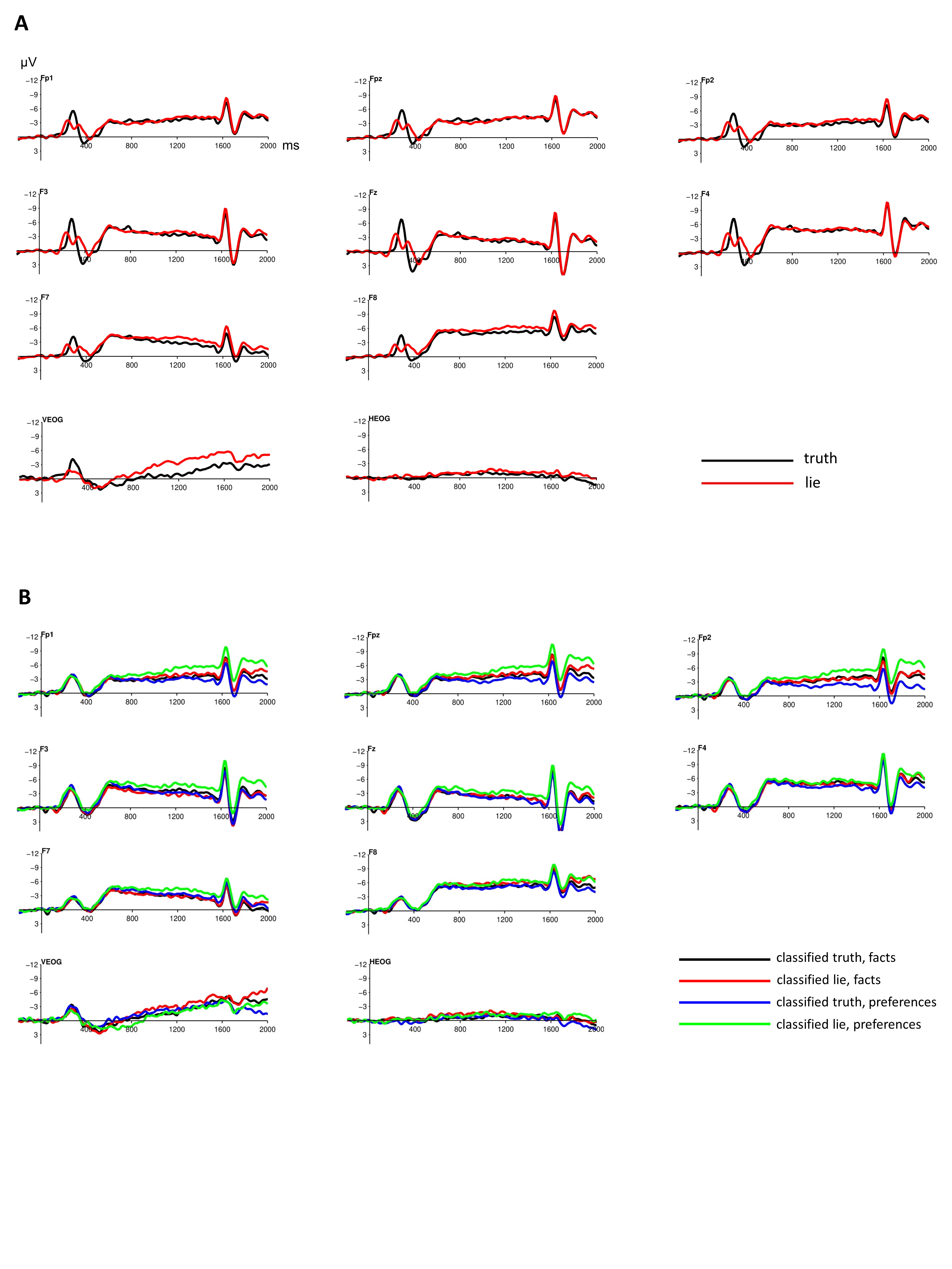

Supplement: FIGURE S1 — (A) Informant stimulus-locked grand average ERP waveforms after the lie/truth cue (trigger 2) at frontopolar/frontal electrodes for the truth and the lie conditions. No significant difference in CNV is present. Differences in early ERP components (N200 and P300) were not analyzed, as we cannot rule out that any differences found between conditions may be driven by the different physical properties of the acoustic stimuli used as truth (sound “Wahrheit”) or lie (sound “Lüge”) cues. Baseline used is -100 to 0 ms. The displayed waveforms were filtered with a 20-Hz low-pass filter. (B) Informant stimulus-locked grand average ERP waveforms after the lie/truth cue (trigger 2) at frontopolar/frontal electrodes for the classified lie versus classified truth conditions, separated for fact and preference statements. Visual inspection suggests that the classified truth versus classified lie difference is driven by preference rather than fact statements, which, however, is not statistically significant. Baseline used is -100 to 0 ms. The displayed waveforms were filtered with a 20-Hz low-pass filter. [file Image_1.TIF]
